# Near-Field Microwave Imaging Method of Monopole Antennas Based on Nitrogen-Vacancy Centers in Diamond

**DOI:** 10.3390/mi15060679

**Published:** 2024-05-22

**Authors:** Xuguang Jia, Yue Qin, Zhengjie Luo, Shining Zhu, Xin Li, Hao Guo

**Affiliations:** State Key Laboratory of Dynamic Measurement Technology, Shanxi Province Key Laboratory of Quantum Sensing and Precision Measurement, North University of China, Taiyuan 030051, China; jiaxuguang10@163.com (X.J.); qytgyx@163.com (Y.Q.); luozzjj@163.com (Z.L.); zhushining1118@163.com (S.Z.); lixinxjx@163.com (X.L.)

**Keywords:** diamond nitrogen-vacancy (NV) center, microwave imaging, monopole antenna, microwave near-field measurement

## Abstract

Visualizing the near-field distribution of microwave field in a monopole antenna is very important for antenna design and manufacture. However, the traditional method of measuring antenna microwave near field distribution by mechanical scanning has some problems, such as long measurement time, low measurement accuracy and large system volume, which seriously limits the measurement effect of antenna microwave near field distribution. In this paper, a method of microwave near-field imaging of a monopole antenna using a nitrogen-vacancy center diamond is presented. We use the whole diamond as a probe and camera to achieve wide-field microwave imaging. Because there is no displacement structure in the system, the method has high time efficiency and good stability. Compared with the traditional measurement methods, the diamond probe has almost no effect on the measured microwave field, which realizes the accurate near-field imaging of the microwave field of the monopole antenna. This method achieves microwave near-field imaging of a monopole antenna with a diameter of 100 µm and a length of 15 mm at a field of view of 5 × 5 mm, with a spatial resolution of 3 µm and an imaging bandwidth of 2.7~3.2 GHz, and an optimal input microwave phase resolution of 0.52° at a microwave power of 0.8494 W. The results provide a new method for microwave near-field imaging and measurement of monopole antennas.

## 1. Introduction

Monopole antennas, known for their simple structure and ease of design and manufacture, have vast applications [1]. Thus, evaluating and characterizing the microwave fields of monopole antennas at all stages, from research and design to manufacturing, commissioning, and maintenance, are important [2]. Traditional near-field measurement techniques for antennas encompass plane scanning, cylindrical scanning, spherical scanning, and polar plane scanning [3]. These methods rely on mechanical displacement structures to measure the microwave near-fields between the probe and monopole antennas [4]. However, achieving more precise microwave near-field data necessitates increasing the sampling points during scanning, resulting in significantly longer measurement times [5,6] and poor response detection effects from the input antenna pulse signal [7]. Typically, these methods require mechanical structures to move the probe for scanning, introducing inevitable random errors during probe movement that are challenging to predict and manage [8,9]. While compensation for probe-induced effects can be achieved through algorithms, it demands extensive experimental data for analysis, substantially augmenting the measurement workload [10]. Placing the metal probe at a distance not less than a few wavelengths from the antenna under test invariably induces coupling between the antenna and the probe, impacting the measurement outcomes [11]. Time-domain measurements may offer advantages over frequency-domain measurements, including faster scan times and more cost-effective measurement devices [12]. Furthermore, in antenna phase testing, traditional methods fail to directly obtain microwave field phase information, necessitating the calculation of missing phase data through other physical quantities or through amplitude data from twice the normal number of sampling points [13], followed by phase restoration using numerical optimization methods. These challenges underscore the limitations of traditional techniques.

Diamond NV centers, renowned for their microwavable spin states and optical readout capabilities at room temperature, have garnered considerable attention in microwave near-field imaging [14]. Concurrently, researchers have demonstrated that NV center-based imaging systems offer several advantages, including a wide operating temperature range, non-destructive testing capabilities, fast imaging, and high sensitivity to microwave fields [15], making them ideal for the near-field imaging of monopole antennas.

The diamond NV color center arises as a defect when a nitrogen atom combines with a vacancy formed in the crystal lattice after replacing a carbon atom in the diamond lattice. There are three kinds of quanta with different spin states in the diamond NV center, and $m_{s}=1$, $m_{s}=0$, $m_{s}=-1$. In the absence of an external magnetic field, the quantum states in $m_{s}=1$ and $m_{s}=-1$ are degenerate states, that is, $m_{s}=\pm 1$. The ground state quantum in the center of NV can be excited by the 532 nm laser and transition to the excited state [16]. A quantum in an excited state will spontaneously radiate energy and transition from the excited state to the ground state. There are two ways to return from the excited state to the ground state: radiative transition and non-radiative transition. The difference between the two is that only the radiation transition can radiate red light with a wavelength of 637 nm. However, the excited quantum in the spin state of $m_{s}=0$ can realize the radiation transition. However, most of the excited quantum states in $m_{s}=\pm 1$ can only achieve non-radiative transitions. Therefore, we call the quantum state $m_{s}=\pm 1$ “dark state” and the quantum state $m_{s}=0$ “bright state”. A quantum state of $m_{s}=0$ in the ground state can be pumped to a quantum state of $m_{s}=\pm 1$ under the action of a specific frequency microwave, to achieve the transformation of “bright state” and “dark state”. The greater the microwave intensity, the greater the number of quanta being pumped, and the lower the fluorescence intensity radiated by the diamond. Thus, the change in fluorescence intensity can reflect the change in microwave intensity [17]. Reflected in the light detection magnetic resonance spectrum is a resonance peak. Therefore, we can characterize the intensity of microwave field by comparing formant peaks. As the microwave field intensity increases, more quanta can absorb enough energy to undergo a quantum state change. That is, when the $m_{s}=0$ quantum state is pumped to $m_{s}=\pm 1$, the quantum number of non-radiative transition increases, the red fluorescence intensity of diamond decreases, and the resonance peak contrast in ODMR spectrum increases. When the microwave power is reduced, the resonance peak contrast in the ODMR spectrum becomes smaller.

**Figure 1:**
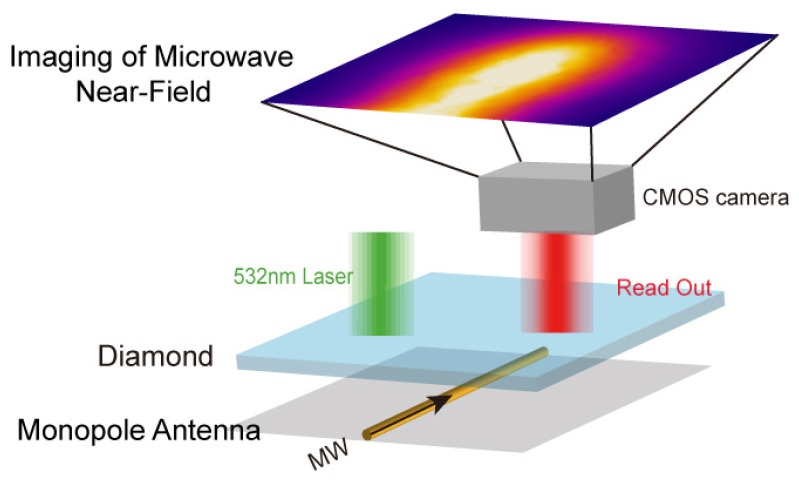
A schematic of the microwave near-field imaging of a monopole antenna. A diamond is positioned above the monopole antenna, with a green laser directed onto its surface. The NV center within the diamond is excited by the laser and microwaves generated by the monopole antenna, leading to red fluorescence. Due to the influence of a non-uniform microwave field, the NV center inside the diamond exhibits a distinct optically detected magnetic resonance (ODMR) spectrum. This ODMR spectrum enables the restoration of the microwave field distribution of the measured antenna. The red fluorescence captured by the CMOS camera generates the image depicting the near-field microwave distribution around the monopole antenna.

In this paper, we introduce a highly efficient technique for the microwave field imaging of monopole antennas utilizing the NV neutral color centers in a diamond. The microwave near-field interactions of monopole antennas, driven by the interplay between microwaves and quantum spin, are elucidated, as depicted in Figure 1. We establish a uniform light experimental system based on a fly-eye lens, supporting both the scanning imaging and diamond-based direct imaging modes. Within the imaging field of view, the light distribution approaches uniformity. Fluorescence intensity images of the diamond are captured using a bandpass filter and a CMOS camera. Subsequently, we devise a microwave field imaging method that computes the microwave field intensity per pixel, leveraging the optically detected magnetic resonance (ODMR) spectral contrast to reconstruct the microwave field image. The experimental system’s validity is confirmed through measurements of the microwave near-field of the monopole antenna. Due to its non-interference characteristics, the absence of electromagnetic interference, and coupling, this imaging method faithfully produces microwave near-field images of the actual equipment. This technique enables researchers to directly, accurately, and rapidly visualize the microwave field distribution of monopole antennas, offering a vital reference for their design, manufacture, and testing.

## 2. Materials and Methods

**Figure 2:**
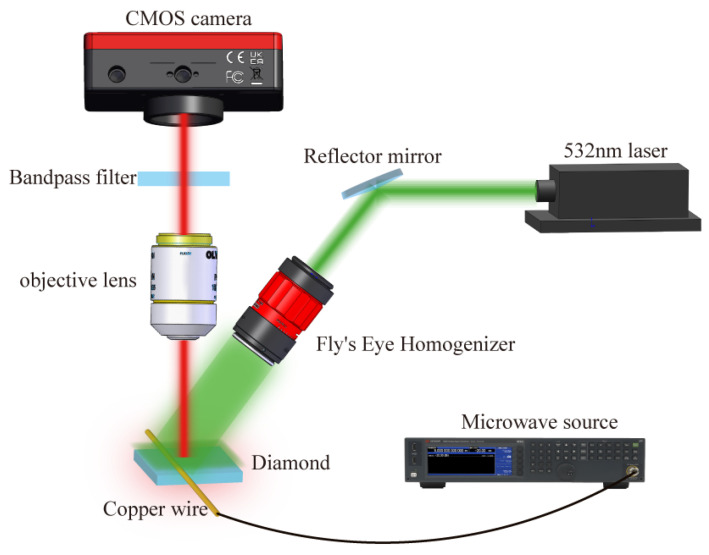
Schematic of the experimental system. We present the setup of the monopole antenna microwave near-field imaging system. At the heart of our measurement setup lies a square diamond chip, with each side measuring 4 mm. To generate a non-uniform microwave field for detection, a copper wire with a radius of 100 μm is placed on the diamond surface and connected to the microwave source. Experimental data are gathered using CMOS cameras.

In Figure 2, the microwave near-field imaging system comprises three main components: an optical system, a microwave system, and a data acquisition system. Within the optical system, the 532 nm laser emits green light, which is successively expanded, collimated, and homogenized before being directed onto the diamond surface. In direct imaging mode, a fly-eye leveling mirror follows the laser collimation to ensure uniform illumination of the diamond surface, achieving a wide field of view [16].

Upon excitation by the 532 nm laser, the NV center emits red fluorescence, collected through the objective lens and filtered before being received by the CMOS camera (LP126 MU). In the microwave system, a fixed microwave antenna beneath the diamond generates the microwave field, with its frequency and power controlled to form various microwave fields [15,17]. Because the unipolar antenna is directly connected to the microwave source through a coaxial cable. Therefore, the output power displayed by the microwave source is the input power of the unipolar antenna. At the same time, we use the output power displayed by the microwave source as the input power of the monopole antenna in the simulation software. The output power of a microwave source is not exactly equal to the input power of a monopole antenna. However, because the coaxial cable and the microwave source can achieve impedance matching, the microwave power loss in the transmission process is very small. The data acquisition system comprises a camera, a pulse generator board, and a workstation. Before fluorescence data acquisition, a synchronization pulse sequence ensures the coordinated operation of the laser, microwave source, and external camera trigger for receiving and chronologically saving red fluorescence signals [18].

Building upon this experimental setup, a microwave field imaging method is implemented, involving four key steps: optical focusing, fluorescence data acquisition, microwave field intensity measurement and characterization, and image reconstruction. Optical focusing establishes the mapping relationship between the diamond’s spatial position and the camera-captured image pixels. The fly-eye homogenizer uniformly illuminates the diamond with a Gaussian laser light, with successful optical focusing confirmed based on a clear diamond image within the camera’s field of view.

**Figure 3:**
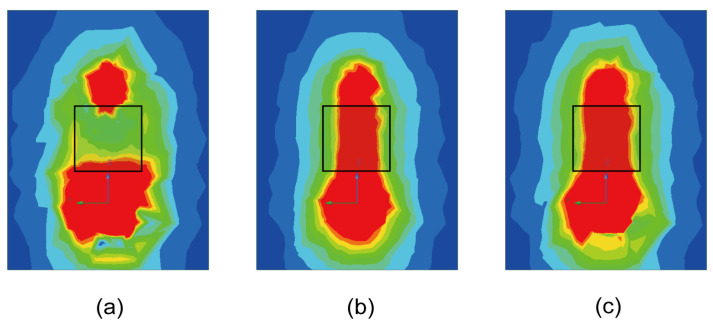
Influence of different dielectric interfaces on microwave field distribution of monopole antennas. The position of the black box in the figure is the interface plane of different media. (**a**) Copper–air (**b**) air–air (**c**) diamond–air. According to the above simulation results, the results of (**c**,**b**) are most similar, indicating that the diamond–air interface plane has little influence on the microwave field.

Before the experiment begins, we need to discuss the effect of dielectric constant on the microwave field distribution of a monopole antenna. In the experimental system, due to the presence of the diamond, the medium around the monopole antenna changed from air to diamond–air, that is, from a single medium to a mixed medium, so its dielectric constant also changed. In the process of microwave propagation, if the critical surface of two different media and the electrical impedance of the propagating medium change, the propagation path will be affected and the reflection phenomenon will affect the microwave field distribution in the near-field area of the antenna [19]. At the same time, in high dielectric constant media, the microwave may decay faster so that the microwave field distribution is more concentrated. In addition, the change in dielectric constant will also affect the optimal operating frequency of the antenna. That is, some microwaves are reflected when they travel through the air to the diamond. In order to further study the influence of diamond–air interface plane on the microwave field distribution of monopole antenna, we set up monopole antenna and mixed media plane in Ansys Electronics 2023 R1. The simulation results are shown in Figure 3. We can find that the diamond–air interface plane has very little effect on the microwave field distribution of the monopole antenna. On the contrary, the copper–air interface plane has a great influence on the microwave field distribution of monopole antennas. It can also be seen that diamond probes have incomparable advantages over traditional metal probes.If the traditional metal probe wants to measure the accurate distribution of microwave field, it must need compensation algorithm, which increases the workload and working cost. However, the reflection of the microwave in the boundary plane is affected by many factors, including the incidence angle, the boundary plane size, the Fresnel reflection coefficient, the microwave field polarization mode, etc., so it is a complicated but meaningful problem [20,21,22].

**Figure 4:**
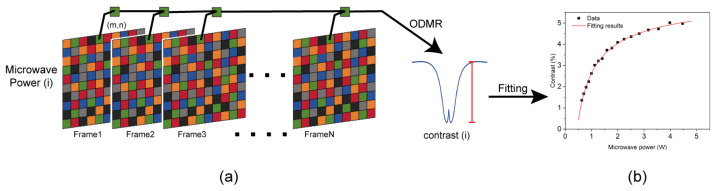
Schematic diagram of fluorescence data acquisition and processing. (**a**), the process begins by dividing the image captured by the camera into multiple units. Subsequently, the CW-ODMR experiment is conducted, wherein the obtained N frames of images are strictly arranged according to acquisition time. Taking any unit (m,n) as an example, the ODMR spectrum of the given unit is computed using the data collected from the unit (m,n) across the N frames. (**b**) illustrates the relationship between the contrast of the ODMR spectrum and microwave power. This relationship is derived by fitting the ODMR spectrum contrast of the same unit under varying power levels with their corresponding powers.

After the optical focusing in the previous step, a definitive relationship is established between the spatial position information of the diamond and the pixels of the image captured by the camera. Subsequently, we introduce a fluorescence data acquisition and processing method, illustrated in Figure 4a. Initially, the data from each pixel of the camera-captured image are extracted; then, these image pixels are averaged. This means that a complete fluorescence image is segmented into multiple units, with the pixel data within each unit combined to form the unit’s data value. Following this, the data from the same position unit in each frame can be sequentially retrieved, both before and after the image acquisition time. This allows for individual calculation and generation of the optically detected magnetic resonance (ODMR) spectrum for each unit. Subsequently, the microwave intensity of each unit is computed separately, thereby enabling the derivation of the microwave field distribution across the entire position of the diamond NV color center probe. The number of pixels within a unit defines the imaging resolution, and thus, we characterize the resolution by the number of pixels M contained in each unit. As M increases, the resolution decreases. However, each unit will encompass more NV color centers, capable of capturing stronger fluorescence signals. This results in an effective enhancement of the signal-to-noise ratio and overall system stability. Hence, M serves as a representation of the performance of the entire imaging system. Following the experiments and considering both imaging speed and quality, the system’s M value is set to 50. Notably, the minimum limit for M is 1. Additionally, as M decreases and the amount of sensing increases, the initialization time and corresponding operational duration of the entire imaging system will increase several times.

Before utilizing this system for reconstructing the microwave field of a monopole antenna, it is imperative to calibrate its ability and accuracy in detecting the microwave field. This involves testing and characterizing the microwave field intensity to establish a basis for subsequent measurements. In the microwave field intensity calibration experiment, an array antenna capable of emitting a uniform microwave field is positioned beneath the diamond chip and linked to the microwave source. The uniform microwave field power emitted by the array antenna is adjusted by varying the microwave source power. Meanwhile, the laser power remains constant as the experiment proceeds, altering only the output power of the microwave source. ODMR spectra under different microwave powers for each sensor unit are obtained by processing multiple sets of collected images. Subsequently, the contrast of the ODMR spectrum is computed and recorded corresponding to the microwave power. Next, the contrast of the ODMR spectrum for each unit is fitted with microwave power data to derive the contrast of the ODMR spectrum for each unit and to establish the microwave power characteristic curve, as depicted in Figure 4b. It can be seen from Figure 4b that within a certain microwave power range, ODMR spectral contrast also increases with the increase in microwave intensity. When the microwave intensity increased to a certain extent, the ODMR spectral contrast increased less with the increase in microwave intensity. When the microwave intensity continues to increase, the ODMR spectral contrast is almost unchanged. This phenomenon is called “microwave saturation”. The reason for the “microwave saturation” phenomenon is that when the microwave power changes, the 532 nm laser power used to excite the quantum ground state to the excited state does not change. And the quantum number that is excited to the excited state is fixed. The microwave power required when all the excited quantums are pumped to $m_{s}=\pm 1$ quantum state is the saturated microwave power. At this time, the quantum number of non-radiative transitions is the largest, and the ODMR spectral contrast is the largest. If the microwave power continues to increase, more quantum is still pumped to the $m_{s}=\pm 1$ quantum state, but the excited quantum number remains unchanged, the quantum number of non-radiative transition remains unchanged, and the ODMR spectral contrast does not increase. That is, the phenomenon of microwave saturation occurs.

During the microwave field reconstruction imaging process, the ODMR spectral contrast obtained from the acquired fluorescence image processing is incorporated into the characteristic curve of each unit. Subsequently, the microwave field intensity of each unit is inversely solved, and the data are summarized based on the position of each unit to generate the microwave field imaging map.

## 3. Results

### 3.1. Monopole Antenna Microwave Field Imaging with Different Microwave Powers

**Figure 5:**
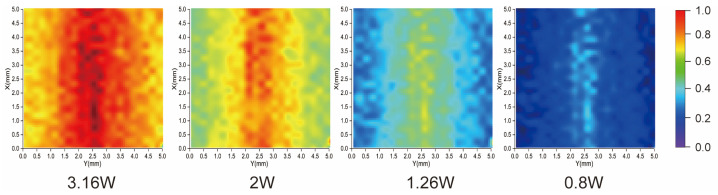
Microwave near-field distribution of monopole antennas at different input powers.

In this experiment, we position the monopole antenna directly beneath the diamond, aligning its position with the diamond’s central axis. Keeping the monopole antenna’s position and the laser power constant, we vary the input power of the monopole antenna. The near-field microwave imaging results under different input powers of the monopole antenna are illustrated in Figure 5.

### 3.2. Monopole Antenna 3D Microwave Field Imaging

**Figure 6:**
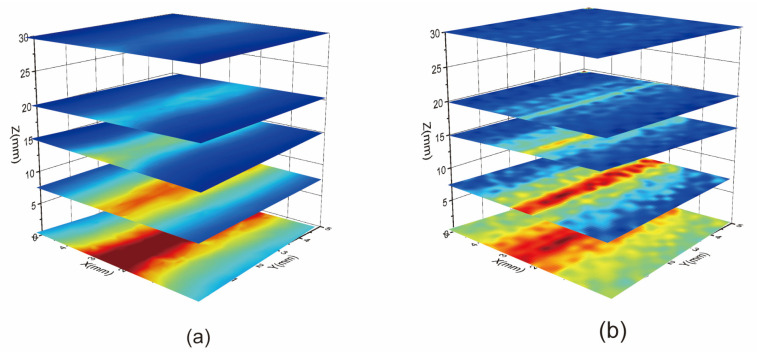
Three-dimensional microwave near-field distribution of monopole antenna. (**a**) Simulation results of three-dimensional microwave field of monopole antenna. (**b**) Experimental results on three-dimensional microwave fields of monopole antennas.

The three-dimensional microwave field of a monopole antenna is constructed using a layered scanning technique. The distance h between the monopole antenna and the diamond is precisely controlled by adjusting the vertical distance of the z-axis of the precision triaxial translation table. In Figure 6, the three-dimensional microwave field of a monopole antenna without a magnetic field is depicted. The microwave field intensity is observed to tend to decrease with an increase in distance h. Moreover, the attenuation coefficients vary in different regions, with larger coefficients near the monopole antennas. To assess the reliability of the experimental results, the microwave near-field of the monopole antenna is simulated in COMSOL Multiphysics 5.6, with the simulation results showing good agreement with the experimental findings.

### 3.3. Monopole Antenna Microwave Field Imaging with Different Input Phases

**Figure 7:**
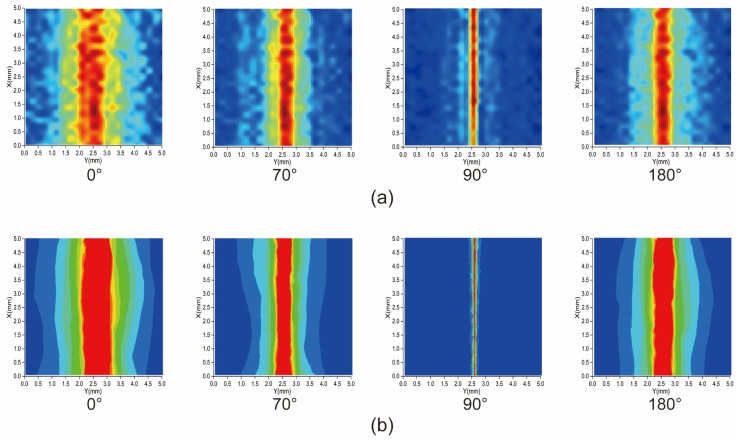
The microwave near-field distribution of a monopole antenna with different input microwave phases. (**a**) The experimental imaging results of the microwave field of the monopole antenna for the same input phases. (**b**) The simulation results of the microwave field of the monopole antenna are depicted for input phases of 0°, 70°, 90°, and 180°.

In this experiment, we maintained the position of the monopole antenna as well as kept the microwave and laser powers unchanged. We varied the microwave phase of the input monopole antenna using a microwave phase shifter. The experimental results are depicted in Figure 7a. Additionally, to validate the accuracy of these results, we simulated the microwave field distribution for different phases of the monopole antenna using Ansys HFSS 2023R1, as illustrated in Figure 7b.

### 3.4. Monopole Antenna Pattern

**Figure 8:**
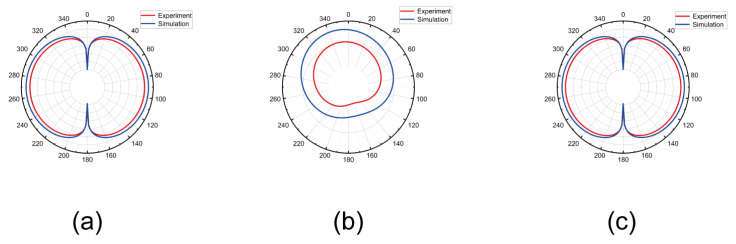
Monopole antenna pattern.In the figure, the red pattern is the monopole antenna pattern deduced from the experimental results, and the blue one is the monopole antenna pattern obtained by simulation in HFSS software. (**a**) XOZ plane antenna pattern. (**b**) XOY plane antenna pattern. (**c**) YOZ plane antenna pattern.

Antenna pattern is an important index of antenna. The antenna pattern is usually measured directly in the far-field region. However, the direct measurement of the antenna pattern requires enough space and can not be measured in a small space such as the laboratory. Antenna near field measurement refers to the measurement of antenna related parameters in the near field region. Compared with the far field measurement, the near field measurement requires less space and faster speed, so the antenna near field measurement data can be obtained quickly. We can convert near-field measurement data into far-field pattern by mathematical transformation [23]. The far field pattern derived from near field measurement data avoids the inconvenience of measuring far field pattern directly, and expands the application scenario of antenna pattern. Antenna design can be optimized in the laboratory with the help of antenna pattern. Figure 8 shows the direction diagram of the monopole antenna. The simulation results are similar to those derived from the experimental data, which proves the correctness of the measurement results of the microwave imaging system, and also expands the application range of the microwave imaging system.

## 4. Discussion

### 4.1. Discussion of Experimental Microwave Field Imaging Results of Monopole Antennas with Different Microwave Powers

From the results of the experiment in Section 3.1, the experimental system evidently effectively detected changes in the input microwave power of the monopole antenna, and the imaging effect of the system varied accordingly. When the input microwave power was relatively low, the contrast in the optically detected magnetic resonance (ODMR) spectrum accurately reflected the microwave power within the linear region of the characteristic curve, theoretically yielding optimal imaging results. However, the actual results fell short of our expectations. This discrepancy arises because the low microwave power led to a diminished ODMR spectrum contrast, making it susceptible to environmental noise, thus resulting in inaccuracies [24]. When the input microwave power is low, it can be seen from Figure 4b that the ODMR spectral contrast measured by the experimental system is also low. In the actual experiment process, due to the influence of environmental factors, the measured ODMR spectrum contains noise interference. When the input microwave power is low, the ODMR spectrum contrast is also low. At this time, the noise does not change, so the signal-to-noise ratio of the test system is reduced, resulting in the experimental results are not consistent with the expected results. Conversely, when the input microwave power was high, the phenomenon of “microwave saturation” appeared. Some imaging regions became distorted.

Aiming at the inaccuracy of experimental results caused by different microwave power, we can use different schemes to reduce the inaccuracy of experimental results. When the microwave power is too low, the environmental noise has the greatest influence on the experimental results. Reducing the ambient noise can significantly improve the signal-to-noise ratio at low microwave power. When the microwave power is too high, we can try to increase the 532 nm laser power to excite more quanta in the ground state, so that the “microwave saturation” state is returned to the unsaturated state. At this time, the relationship between microwave power and ODMR spectral contrast is in the linear region, avoiding the influence caused by the nonlinear region.

### 4.2. Discussion of Experimental Monopole Antenna 3D Microwave Field Imaging

In the results of the experiment in Section 3.2, the microwave field intensity was observed to decrease as distance h increased. Additionally, varying attenuation coefficients were noted across different regions, with larger coefficients observed near the monopole antennas. To assess the reliability of these experimental findings, the microwave near-field of the monopole antenna was simulated using COMSOL Multiphysics 5.6. Remarkably, the simulation results closely aligned with the experimental observations, underscoring the validity and accuracy of the experimental results.

### 4.3. Discussion of Experimental Monopole Antenna Microwave Field Imaging with Different Input Phases

**Figure 9:**
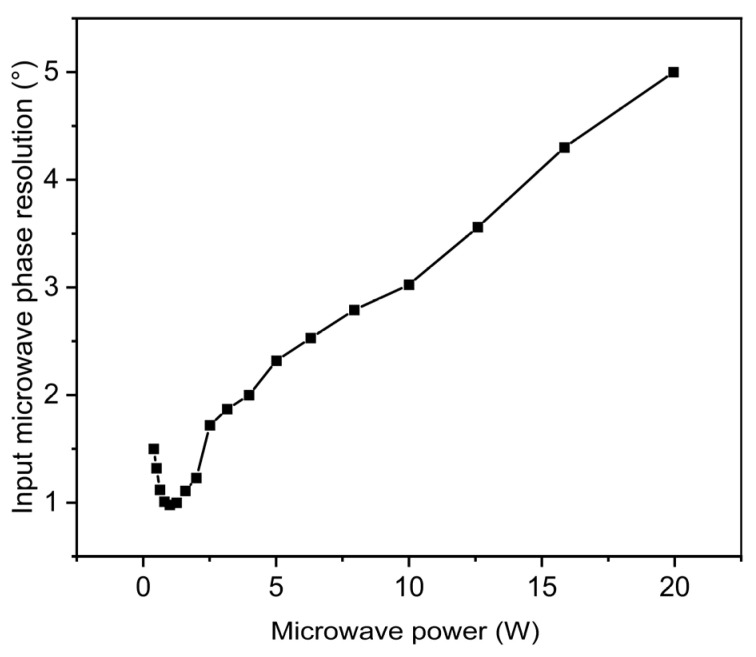
Input microwave phase resolution versus input microwave.

From the results of the experiment in Section 3.3, when the microwave phase was 90°, the microwave field distribution on the surface of the monopole antenna was extremely inhomogeneous and was only in a very small area on the surface of the monopole antenna. However, under the same conditions, the microwave field distribution of the experimental results was larger than that of the simulation results. This is because imaging such a small range (~0.01 mm) of microwave field attenuation during the actual experiment is impossible due to the limitations in the resolution of the spin imaging system itself. Because the small range of microwave field attenuation requires high imaging resolution to show these details. If the attenuation size of the microwave field in a small area is less than the resolution limit of the experimental system, the imaging results cannot show the details here. We obtained Figure 9 by varying the microwave input power. From Figure 9, the resolution of the monopole antenna microwave near-field imaging system for the input microwave phase can be seen to vary with the microwave power. When the microwave power was too high or too low, its resolution became lower. In this experiment, we obtained the best resolution of 0.52° at an input microwave power of 0.8494 W. Additionally, the microwave field in this experiment was in the dynamic phase. The phase change was more complicated compared with the power change in the unipolar microwave field. Because in the near-field microwave field imaging of the monopole antenna, as long as the microwave power is in the linear region shown in Figure 4b, the near-field microwave field imaging of the monopole antenna can be accurate. For dynamic phase tracking, although the phase change is as continuous as the microwave field change. However, if the phase change is less than the phase imaging resolution, it is impossible to achieve accurate phase imaging and phase tracking. In addition, in order to achieve phase tracking, phase imaging needs to be implemented in a short time. Better phase tracking can be achieved only by realizing faster phase imaging speed. In addition, we can infer the microwave power variation of a monopole antenna from any unit value in the microwave field imaging diagram. However, the phase change in the monopole antenna requires image recognition through machine learning to accurately judge the phase change in the microwave.

Ambient noise is always an important factor in phase-tracking experiments of monopole antennas. The higher the ambient noise, the lower the signal-to-noise ratio of the system, resulting in lower precision of phase measurement. Especially for the measurement of small changes in phase, it is easy to be disturbed by environmental noise, so the next step can be carried out in a vacuum, constant temperature, and an active vibration isolation platform environment.

Through this experiment, we achieved dynamic phase tracking of a monopole antenna, i.e., judged the working phase of the monopole antenna according to its microwave field distribution.

### 4.4. Comparison between Diamond Based near Field Microwave Imaging System and Traditional Antenna Measurement System

**Figure 10:**
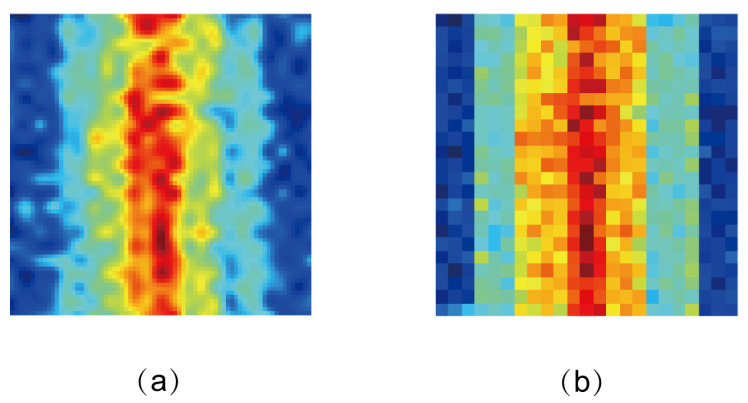
Microwave field imaging results of monopole antennas with different measurement methods under the same microwave power. (**a**) Near-field microwave field results of monopole antenna based on quantum spin measurement method of diamond NV centers. (**b**) Near-field microwave field imaging results of monopole antenna based on traditional measurement methods.

In order to show the difference between traditional antenna measurement methods and quantum spin microwave field imaging methods, we use the same monopole antenna to perform experiments at exactly the same input microwave intensity. When imaging the microwave field of a monopole antenna using traditional antenna measurement methods, the monopole antenna is placed on a high-precision displacement platform and a probe is used as the probe. The probe moves in an S-shaped path. The microwave field distribution diagram is composed of the collected data according to its coordinates, as shown in Figure 10b. Figure 10a shows the measurement results obtained using the quantum spin microwave field imaging method. Based on the measurement results, we found that the quantum spin microwave field imaging method has higher resolution and more detail. However, the traditional measurement method will lose some data and details because of the point-by-point scanning method.

Table 1 shows the measurement time required by the two measurement methods at different resolutions. Because the quantum spin microwave field imaging method can achieve different resolutions by changing the data processing parameters, the measurement time is fixed regardless of the resolution in the measurement step. However, the traditional measurement method needs to change the scanning points and paths to achieve different resolutions, and the measurement time increases with the increase in resolution. When the resolution is too high, the distance between the two points of the point-by-point scanning is less than the diameter of the probe, then the probe will appear repeated points or missing points in the scanning process, which seriously affects the microwave field imaging effect. Therefore, when the resolution in Table 1 is 100 × 100 and 150 × 150, the traditional measurement method cannot obtain effective data.

## 5. Conclusions

In recent years, the miniaturization of monopole antennas has been the goal of people’s pursuit. However, microwave near-field distribution imaging for micro-monopole antennas has been a long-standing demand. Traditional measurement methods have obvious shortcomings in non-invasiveness, measurement efficiency and imaging accuracy [25]. Although these deficiencies can be compensated for using the compensation algorithm to a certain extent, it undoubtedly increases the preparation time of measurement work and affects the measurement efficiency of the antenna.

In this paper, a near-field microwave imaging method based on a diamond NV center was proposed. Compared with traditional measurement methods, this quantum method uses a diamond as the sensing unit, which can be placed very close to the surface of the monopole antenna. Because the sensing element is an atomic defect inside the diamond, this method is less invasive. This paper also introduced a data processing method for a microwave near-field imaging system based on monopole antennas. In this experiment, we characterized the three-dimensional microwave field radiated by the monopole antenna under varying input power levels. Utilizing layered scanning technology, we accurately reconstructed the three-dimensional microwave field of the monopole antenna. Furthermore, we investigated the impact of input phase variations on the microwave near-field of the monopole antenna, adjusting the input phase using a microwave phase shifter. Subsequently, we used this system to image the microwave field of the monopole antenna under different input phases. Remarkably, these imaging results closely match the simulation data, underscoring their excellent reliability. This imaging technique promises to offer valuable insights for the design, manufacturing, and testing of monopole antennas. In future research endeavors, we aim to develop a microwave near-field-focused structure for the monopole antenna, enabling remote imaging of a microwave field. This advancement will provide a novel solution for the non-contact and wide-field imaging of microwave fields.

## Figures and Tables

**Table 1:** Measurement time for microwave imaging with different resolutions.

Imaging Resolution	Diamond Method	Traditional Method
10 × 10	20 s	10 s
50 × 50	20 s	65 s
80 × 80	20 s	112 s
100 × 100	20 s	\
150 × 150	20 s	\

## Data Availability

The original contributions presented in the study are included in the article, further inquiries can be directed to the corresponding author.
